# Use of compressed sensing to expedite high-throughput diagnostic testing for COVID-19 and beyond

**DOI:** 10.1371/journal.pcbi.1010629

**Published:** 2022-10-24

**Authors:** Kody A. Waldstein, Jirong Yi, Myung Cho, Raghu Mudumbai, Xiaodong Wu, Steven M. Varga, Weiyu Xu

**Affiliations:** 1 Interdisciplinary Graduate Program in Immunology, University of Iowa, Iowa City, Iowa, United States of America; 2 Department of Electrical and Computer Engineering, University of Iowa, Iowa City, Iowa, United States of America; 3 Department of Electrical and Computer Engineering, Penn State Behrend, Erie, Pennsylvania, United States of America; 4 Department of Microbiology and Immunology, University of Iowa, Iowa City, Iowa, United States of America; 5 Department of Pathology, University of Iowa, Iowa City, Iowa, United States of America; University of Pittsburgh, UNITED STATES

## Abstract

The rapid spread of SARS-CoV-2 has placed a significant burden on public health systems to provide swift and accurate diagnostic testing highlighting the critical need for innovative testing approaches for future pandemics. In this study, we present a novel sample pooling procedure based on compressed sensing theory to accurately identify virally infected patients at high prevalence rates utilizing an innovative viral RNA extraction process to minimize sample dilution. At prevalence rates ranging from 0–14.3%, the number of tests required to identify the infection status of all patients was reduced by 69.26% as compared to conventional testing in primary human SARS-CoV-2 nasopharyngeal swabs and a coronavirus model system. Our method provided quantification of individual sample viral load within a pool as well as a binary positive-negative result. Additionally, our modified pooling and RNA extraction process minimized sample dilution which remained constant as pool sizes increased. Compressed sensing can be adapted to a wide variety of diagnostic testing applications to increase throughput for routine laboratory testing as well as a means to increase testing capacity to combat future pandemics.

## Author summary

The rapid spread of COVID-19 highlighted the need for testing approaches that are rapid and accurate while reducing the use of critical testing reagents when resources are scarce. One method to increase testing throughput and reduce material usage is to pool samples prior to testing. With this method a single negative result indicates all samples within the pool are negative. However, the efficiency gains from pooling samples decreases when the infection prevalence rate is high, as with the COVID-19 pandemic, where many pools will return positive test results. In this study, we present a novel mathematical approach to pooled testing based on compressed sensing theory allowing us to accurately identify infected samples within a pool at high prevalence rates. Experimentally, we validated our compressed sensing method in a coronavirus model system as well as with primary human COVID-19 nasal swab samples. Using our method, we were able to reduce the number of tests required by 69% while identifying infected samples with 100% accuracy. Our compressed sensing pooled testing method exhibited high accuracy and reproducibility and offered several advantages including the conservation of vital supplies and increased throughput that may facilitate a more rapid response to future pandemics.

## Introduction

The rapid spread of SARS-CoV-2 worldwide placed a significant burden on diagnostic testing and public health to provide fast and accurate testing strategies. The number of COVID-19 tests being performed each day has increased 8-fold since testing reagents became widely available. In the United States, an average of 1–2 million COVID-19 quantitative reverse transcription polymerase chain reaction (qRT-PCR) tests were performed each day in November 2020-May 2021, August 2021-October 2021, and December 2021-February 2022. To date, the total number of tests performed in the US is nearly 1 billion with a daily average still ranging from 50,000 to over 1 million [1–3]. Additionally, multiple new and more infectious variants of COVID-19 continue to emerge worldwide harboring genetic mutations significant enough to result in breakthrough infection causing concern for current vaccine formulations [4–8]. Continued testing and screening remain critically important to minimize virus spread, thus the development of innovative strategies and techniques to increase testing capacity without reducing the accuracy and efficacy of testing is crucial.

A traditional method to increase testing capacity is by pooling samples as opposed to conducting individualized testing, known as “group testing” [9–11]. When the prevalence rate is low within the population, the majority of samples will test negative, thus a single negative result indicates that all patients within that pool are negative. However, the ability to accurately identify the status of individual samples using this method diminishes quickly as the prevalence rate increases [12–15]. Current CDC guidelines require subsequent individual testing of all patients within a pool if the pool is positive [16]. Throughout the SARS-CoV-2 pandemic, locally high prevalence rates of >10% have been observed repeatedly with some areas surpassing 30% multiple times during recent waves of infections caused by newly emerging virus variants [17–19]. These high prevalence rates are well beyond the capacity of traditional pooling methods as many pools will be positive requiring additional individual testing and inevitably increasing the number of tests required. More sophisticated pooling efforts have been developed and validated during the pandemic though the accuracy and effectiveness of these new approaches decreases as the prevalence rate rises, highlighting the need to develop new approaches that can be used at high prevalence rates during the current and future pandemics [13,16,20–23].

In this study, we present a novel and innovative pooling protocol based on compressed sensing theory utilizing mathematically-derived mixing matrices and decoding algorithms to accurately identify positive patients within pools at high prevalence rates. The application of compressed sensing in signal analysis is widely documented in the literature. In the biological sciences, compressed sensing has been employed in the identification of rare genetic alleles and signal acquisition in fluorescence microscopy [24–27]. The compressed sensing theory indicates that reconstruction of a sparse signal can be achieved with fewer measurements than linear signal processing. The assumption is that the majority of signal measurements will be either zero or irrelevant. This aspect of compressed sensing and signal analysis directly applies to diagnostic testing, even in cases of high prevalence.

Additionally, we propose a new approach to pooled testing in which the viral load of each individual sample within a pool can be quantified [28–30]. Secondary to the power of compressed sensing to reduce the number of measurements needed, the main objective is to provide an estimate of the original signal. In the case of pooled testing, that translates to a real number determination of individual patient viral loads within a pool, not simply the overall viral load of the pool. Our proposed approach works both in a non-adaptive setting, where only one qRT-PCR run is performed for a group of samples, and in an adaptive setting, where additional qRT-PCR runs can be requested based on earlier testing results. We also employ a modified RNA extraction process in which the patient swab samples are pooled prior to RNA extraction allowing the sample to be concentrated thus minimizing sample dilution and the possibility of false negative results. This approach has shown high accuracy and reproducibility at prevalence rates >10% with various sample sizes using an experimental mouse coronavirus, mouse hepatitis virus strain 1 (MHV-1), as well as human nasopharyngeal patient samples containing SARS-CoV-2 RNA.

## Results

We consider the problem of estimating the viral load of a population with *N* individuals $x\in R_{+}^{N}$ from their pooled testing results in *n* pools. The pooling protocol is modeled by a mixing matrix $A\in R_{+}^{n\times N}$ which is a composite of the participation matrix *P*∈{0,1}^*n*×*N*^ and the viral load adjusting matrix $W\in R_{+}^{n\times N}$, i.e., *A* = *P*⊗*W* where ⊗ denotes element-wise multiplication, i.e., *A*_*ij*_ = *P*_*ij*_*W*_*ij*_. We denote by $b\in R_{+}^{n}$ the viral load of all the pools after the mixing but before the qRT-PCR amplification, i.e., *b* = *Ax*. The value of *b* can be determined by the Ct value readout of the pools after the qRT-PCR. The *i* -th pool is claimed to be positive or *p*_*i*_ = 1 if *y*_*i*_∈*Ω*, and negative or *p*_*i*_ = 0 if *y*_*i*_∉*Ω* where *Ω* is the range of Ct value, e.g., *Ω* = [12,34], and *p*∈{0,1}^*n*^ is the status of all the pools. This relation can be modeled by an indicator function *I*_*Ω*_ as follows:

$$p_{i}=I_{\Omega }(y_{i})=\left\{ \begin{array}{c} 1,if\;y_{i}\in \Omega ,\\ 0,if\;y_{i}\notin \Omega . \end{array}\right.$$

The quantitative relation between *b* and *y* can be obtained via interpolation, i.e., *b* = *f*^−1^(*y*) where *f*^−1^ is the element-wise function for mapping the Ct value to viral load, and it is obtained via interpolation, i.e., *b*_*i*_ = *f*^−1^(*y*_*i*_). For the sake of reducing the false negative at the cost of more later tests, a technician can be conservative enough to mark positive results for mixtures although they have moderately large Ct values for which negative results can be assigned when the criterion is relaxed. We model this process as *b*_*i*_ = *f*^−1^(*y*_*i*_+*η*_*i*_).

**Fig 1:**
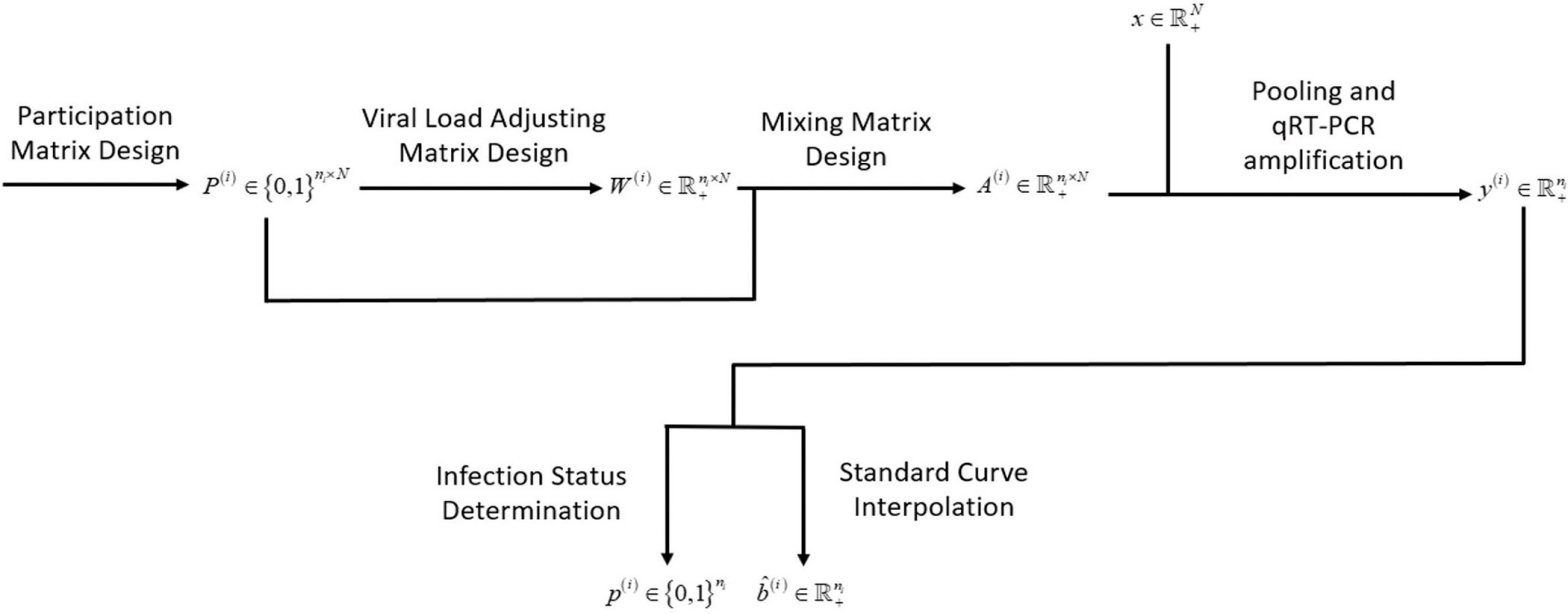
Mathematical framework for pooling in the *i* -th stage in a multiple-stage pooling strategy.

In practice, multiple stages of pooling may be required to achieve a balance between efficiency and accuracy. In this scenario, we have a group of participation matrices, i.e., $P^{\left(i\right)}\in {\left\{0,1\right\}}^{n_{i}\times N},i=1,2,\cdots ,$ viral load adjusting matrices, i.e., $W^{\left(i\right)}\in R_{+}^{n_{i}\times N},i=1,2,\cdots ,$ and mixing matrices, i.e., $A^{\left(i\right)}\in R_{+}^{n_{i}\times N},i=1,2,\cdots ,$. A visual illustration of the above mathematical modeling framework of the pooling procedure in the *i* -th stage is presented in **Fig 1.**

Our goal is to decode the status of all the individual samples, i.e., positive (meaning that a sample contains viral RNA) or negative (meaning that a sample does not contain viral RNA), and the viral load of each sample. The problem can be solved under the compressed sensing framework by finding the solution to the following *L*_1_ optimization problem, *min*_*x*_‖*x*‖_1_, such that *f*^−1^(*y*) = *Ax*, under the assumption that *x* is sparse, or *min*_*x*_‖*x*‖_1_, such that *f*^−1^(*y*+*η*) = *Ax* where *η*∈ℝ^*n*^ characterizes the noise or reading error occurring in the readout of Ct values [28, 30].

Though the accuracy outcomes from solving the above optimization problems are favorable when *N* is large, that is not optimal for keeping the complexity of the mixing process low in clinical virus testing. Therefore, we will focus on the cases where *N* is small, i.e., *N* = 7,15,31,40, which differs from the case in compressed sensing where the values of *N* and *n* are large.

### Mixing matrix design

The design of mixing matrix *A*∈ℝ^*n*×*N*^ involves a participation matrix and a viral load adjusting matrix. The participation matrix *P*∈{0,1}^*n*×*N*^ is a binary matrix, and its elements indicate the participation of individual samples in different pools, i.e., the *i* -th individual sample participates in the *j*-th pool if *P*_*ji*_ = 1, and it does not appear in the *j*-th pool if *P*_*ji*_ = 0. The elements of the viral load adjusting matrix $W\in R_{+}^{n\times N}$ indicate the adaptation or scaling factor of the viral load (copy number/volume) of individual samples that are used in different pools due to the pooling procedure where $R_{+}^{n\times N}$ is the set of positive real numbers. For example, the viral load of *i*-th individual sample added to the *j*-th pool is diluted by 4 times if $W_{ji}=\frac{1}{4}$.

In our laboratory experiments, we utilized an MHV-1 coronavirus model system in which we can concentrate and fix the sample dilution. In our primary human RNA samples containing SARS-CoV-2 viral RNA, the samples were supplied as pre-extracted RNA and could not be concentrated after pooling [31–36]. Thus, the design of both the participation matrix and the viral load adjusting matrix differs for these two virus models illustrating our system’s ability to adapt to real-world situations. In our experiments with MHV-1, the participation matrices are obtained by constructing the parity check matrices. There is a one-to-one correspondence between the participation matrix *P* and the mixing matrix *A*, we will refer them alternatively in the subsequent sections.

### Parity check matrix and fixed dilution

We now introduce how the participation matrix *P* and the viral load adjusting matrix *W* are designed for MHV-1 with a small population size *N*, i.e., *N* = 7, 15, and 31, and a low prevalence rate. In this scenario, there is approximately one infected individual within *N =* 7, 15, or 31. From information theory, we know that the parity check matrix for Hamming codes can guarantee the identification of one error in codewords or the identification of the parity check matrix column which corresponds to the error in the codewords [37]. In virus testing, such a matrix can guarantee the identification of the positive sample within all of the tested samples. This exactly fits our need for the laboratory experiments with MHV-1 using a small population size and low prevalence rate, and we can use such parity checking matrices as the participation matrices.

**Fig 2:**
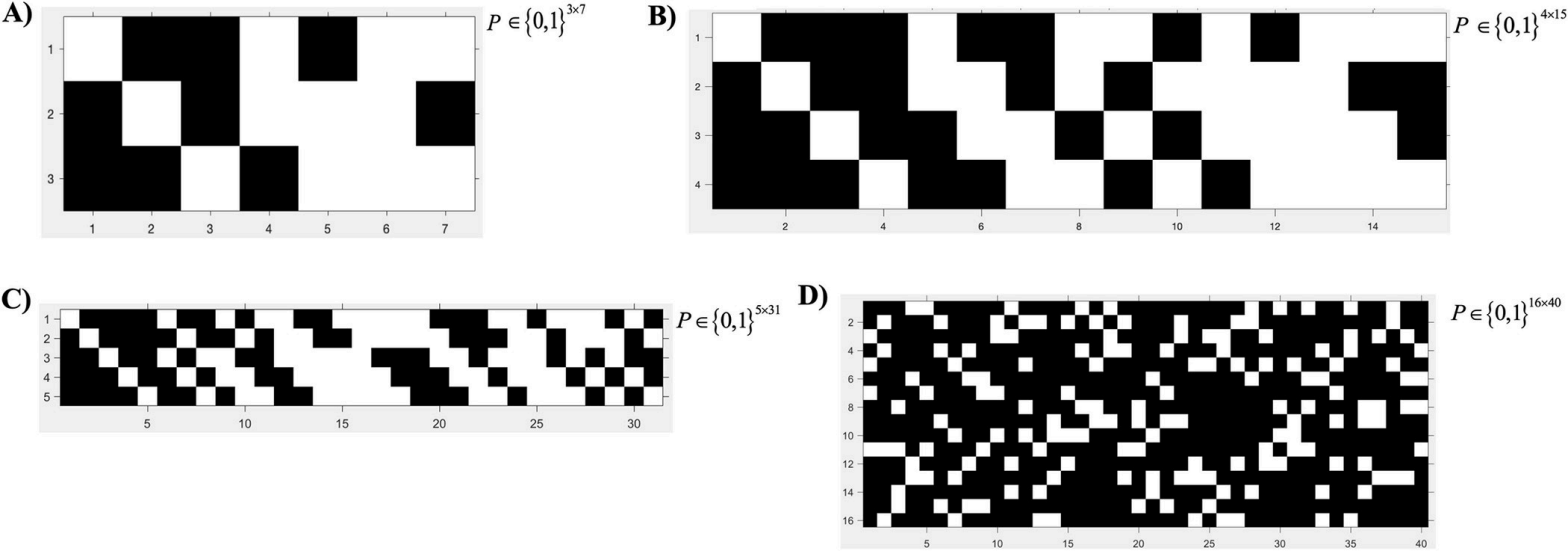
Optimized group testing mixing matrix design. **(A-C)** Hamming code parity check pooling matrix design for *N* = 7, 15, and 31. **(A)** *N* = 7 numerical matrix with 3 pools (3x7). **(B)** *N* = 15 numerical matrix with 4 pools (4x15). **(C)** *N* = 31 pixel matrix with 5 pools (5x31). **(D)** Bipartite pooling matrix design optimized for high *N* and prevalence rates. *N* = 40 pixel matrix with 16 pools (16x40). **(A,B,C,D)** White pixel indicates a sample included in the pool. Black pixel indicates a sample not included in pool.

The construction of such parity check matrices can be described as follows. We let *N* = 2^*n*^−1, and the columns of *P* are simply all the nonzero binary sequence of length *n*. For example, when *n* = 3, each column of *P* has 3 elements. Since each element of the column can be either 0 or 1, we have totally 2^3^ different columns. After removing the column with all zero elements, the rest *N* = 2^3^−1 columns are used to form *P*. As we consider *N* = 7, 15, and 31, the corresponding participation matrices are shown in **Fig 2A–2C**.

In our laboratory experiments with MHV-1, since we take 5 μL from each individual sample to form the sample pool which is then concentrated to a volume of 20 μL regardless of the number of individual samples participating in the pool implying that the viral load for an individual sample within a pool is ¼ of its original viral load. Thus, we can design the allocation matrix as follows:

$$W_{ij}=\left\{ \begin{array}{l} 1/4,P_{ij}\neq 0\\ 0,P_{ij}\neq 0 \end{array}\right.,i\in [n],j\in [N].$$

### Bipartite graph matrix and unfixed dilution

Though these parity check matrices are easy to construct, it cannot scale up for large *N* or high prevalence rates since such parity check matrices can only guarantee the identification of one positive sample, while high *N* or prevalence rates can result in more than one positive sample in the population. Another consequence of a large *N* is the high number of nonzero elements in the participation matrix, which results in increased complexity during laboratory experiments. This motivates us to design participation matrices which can not only succeed in scenarios where positive samples are present, but also have low complexity as indicated by the number of nonzero elements in the participation matrix. We propose to use the binary matrices constructed using a bipartite graph as the participation matrices [38,39]. A bipartite graph is a graph *G*(*L*, *R*, *E*) with two sets of vertices, i.e., left vertices *L* and right vertices set *R*, and edges only exist between vertices from different vertex sets. For a given bipartite graph, each vertex *i* in *L* corresponds to a column in a binary matrix *P*, and each vertex *j* in *R* corresponds to a row in the matrix. The matrix has element *P*_*ji*_ = 0 if there is no edge between *i* and *j*, or *P*_*ji*_ = 1 if an edge exists. As we know, each variable (sample) is assigned to a subset of measurements (pools). Intuitively speaking, we select a different subset of pools for each variable in a way such that every possible k infected samples are connected to a sufficiently distinct set of measurements. In this way, by looking at the measurement results (for example, which pools are positive), one can inversely figure out which samples are infected. More precisely, in compressed sensing, we would like to pick the subset of pools for each variable in a way such that every 2k column vectors of the corresponding 0–1 measurement matrix are linearly independent [28,29,38,39]. We designed our measurement matrix or the bipartite graph according to the design of expander graph which has nice theoretical performance guarantees [39]. In this approach, we assign each sample to l randomly chosen pools. According to coding-theoretic results, such resulting bipartite graphs will correspond to a satisfactory measurement matrix with high probability if the number of pools is large enough [39]. After generating the measurement matrix from such procedures, we use our previously designed optimization methods from Cho M, et al. 2018 to verify the required algebraic conditions are satisfied for this generated matrix [38]. In fact, after testing many randomly generated matrices using this approach, we find that the bipartite graph shown in Fig 2D (having 4 pools per sample) satisfies the required algebraic condition for distinguishing 2 Infected samples among 40 samples, with theoretical performance guarantees [38]. In practice, we find that this matrix works very well, often greatly outperforming the (conservative) performance guarantees we computed, meaning the algorithm figures out which samples are infected even when the number (k) of infected samples is bigger than the guaranteed sparsity 2. We use the above approach to construct a well-designed binary matrix *P*∈{0,1}^16×40^ for our SARS-CoV-2 experiments with each column having only 4 nonzero elements as shown in **Fig 2D**. In our case, *R* is the set of pools, and *L* is the set of individual samples.

For SARS-CoV-2 virus testing in our laboratory experiments, since equal volumes of samples participating in a particular pool are mixed together without sample concentration, the viral load for each individual sample in the mixture is scaled down by the number of participants. Thus, the allocation matrix can be designed as:

$$W_{ij}=\left\{ \begin{array}{l} \frac{1}{\sum_{j=1}^{N}P_{ij}},P_{ij}\neq 0\\ 0,P_{ij}=0 \end{array}\right.,i\in [n],j\in [N].$$

### Sample pooling

**Fig 3:**
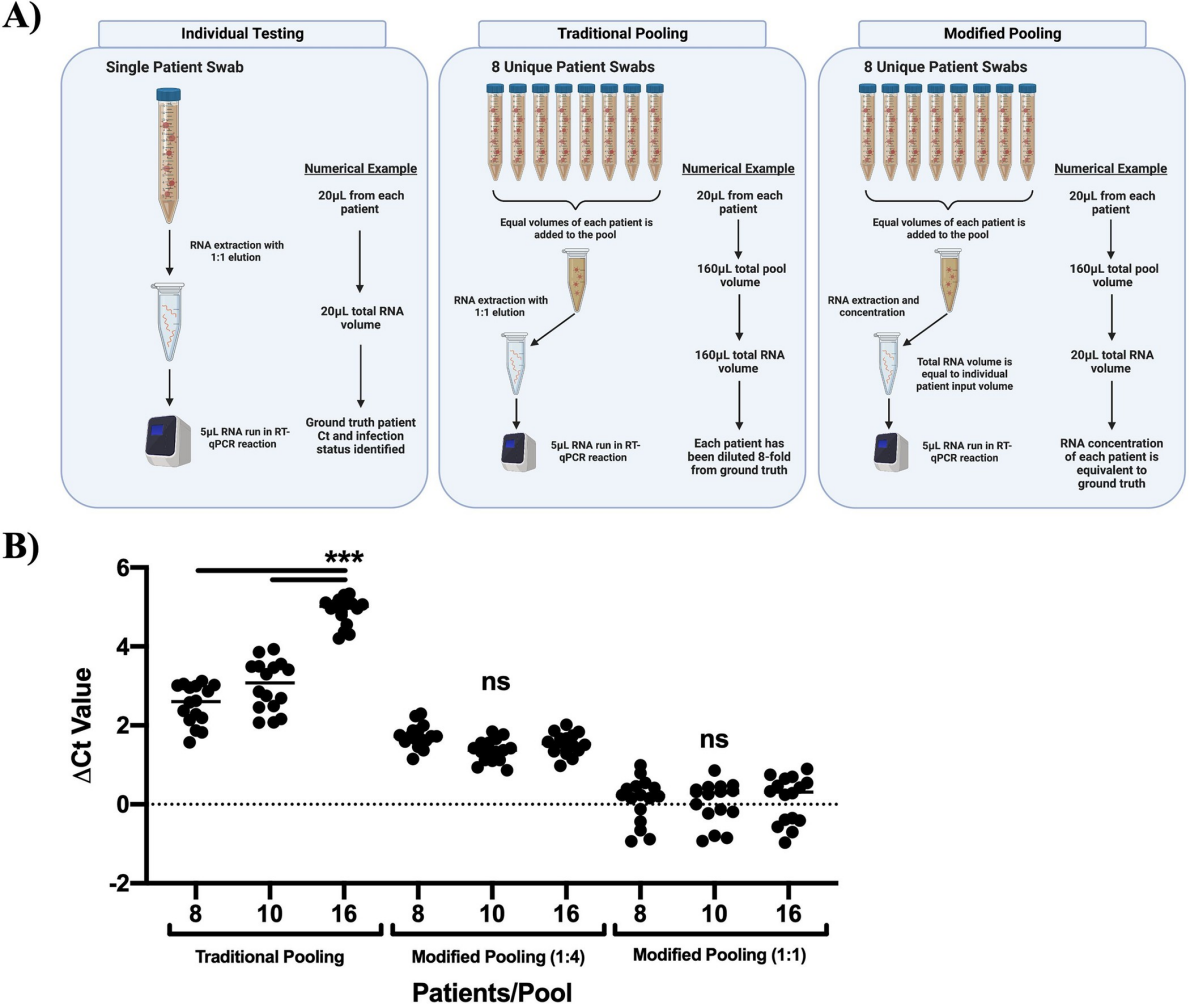
Modified pooling protocol eliminates dilution effect of group testing. (A) RNA extraction and qRT-PCR workflow in individual testing, traditional pooling (group testing), and the modified pooling protocol. Numerical examples are theoretical to display dilution effect and can be scaled to individual diagnostic testing facility protocols. (B) MHV-1 was used to generate individual samples of various viral loads (1x10^9^-1x10^2^ copy number/qRT-PCR reaction). qRT-PCR was performed on each sample to develop ground truth Ct values. Samples were then used in various pool sizes in traditional pooling and in the modified pooling protocol. Increases in sample Ct values from the ground truth values were calculated and plotted as ΔCt Value. Data are presented as the mean ± SEM from two combined independent experiments (*n =* 16). For statistical analysis a one-way ANOVA with a Tukey’s post hoc test was performed. ***p<0.001, ns = not significant. Created with BioRender.com.

In many group testing processes, patient samples are pooled after RNA extraction or the total pool volume dictates the RNA elution volume. In both cases, this means the fold dilution of each sample is dependent on the total number of samples within a pool. Thus, as the number of patients pooled increases, the sample becomes more dilute, significantly increasing the probability of a false negative test result. This phenomenon has required pools to remain small, usually less than 5 patients per pool [14,15,23,40]. To reduce the dilution effect of pooling, a modified RNA isolation protocol was developed using TRIzol phenol/chloroform that can be more broadly applied to RNA extraction kits and automated systems such as the KingFisher [41]. With this method, patient samples are pooled prior to RNA extraction. After the isopropanol precipitation and ethanol step, the pelleted RNA can be significantly concentrated by reducing the final volume of water used to solubilize the RNA thus minimizing the potential impact of sample dilution **(Fig 3A)**.

To test the dilution effect of traditional pooling on qRT-PCR Ct results as compared to our modified RNA extraction protocol, we utilized the widely used murine coronavirus MHV-1 as a model system [31–36]. Using a MATLAB-derived computational script, we pseudo-randomly generated simulated samples based on a Ct value range of 12–34 cycles. These experimental parameters were chosen from current CDC testing guidelines and growing evidence that individuals with viral loads corresponding to a Ct value of 34 and above are likely non-infectious and/or not reliable to diagnose positive patients [42–45].

As expected, samples pooled by traditional group testing exhibited a significant impact on the Ct value resulting in signal dilution (**Fig 3B)**. However, the dilution effect was minimized or eliminated in the modified RNA extraction protocol. (**Fig 3B)**. Importantly, the ΔCt was consistent among all pools regardless of the number of samples indicating the pool size could be significantly increased without causing further sample dilution. These results suggest that the dilution caveat of traditional group testing can be minimized by our modified extraction protocol which could be implemented using automated RNA extraction devices in clinical labs. Patient RNA samples can also be concurrently extracted individually and banked if repeat testing is required. This approach provides a standard dilution effect that is consistent regardless of either the pool size or the volume which significantly simplifies downstream computation and decoding while reducing the chance of a false negative result.

### Viral load decoding with success certificate

We next developed a decoding algorithm which decodes each sample’s viral load from testing results of pooled samples. A unique feature of our decoding algorithm is the decoding success certificate it provides: assuming that the PCR instrument testing readings are accurate, our decoding protocol and algorithm can guarantee that the decoding results reveal the only set of positive samples that fit the testing results, and thus guarantee correctly identifying all the positive and negative samples.

We want to emphasize that in the virus testing practice, we will only have the Ct value data *y*, and the qualitative data *p* which is obtained from the Ct value. So, if there is no error, a pool has positive testing results, i.e., *p*_*i*_ = 1 if and only if there is at least one positive element of *x* participating in the *i*-th pooling test. The goal of viral load decoding is to decode the viral load vector *x* from Ct value data *y*. Thus, we end up with solving for *x* using the under-determined measurement systems, *i*.*e*., *f*^−1^(*y*) = *Ax* where *f*^−1^ is the inverse function of *f*. Note that in virus testing, the Ct value is first obtained from the qRT-PCR, and then used for interpolating the virus load *f*^−1^(*y*) for each pool. Since we reduce the number of tests used, this is often an underdetermined system. However, since the viral load vector *x* is often sparse (meaning only a small number of samples are positive), we are motivated to borrow techniques from compressed sensing to solve for the sparse *x*. In compressed sensing literature, the problem is usually solved by the basis pursuit approach *min*_*x*_‖*x*‖_1_, such that *f*^−1^(*y*) = *Ax* under the assumption that *x* is sparse [28,30]. However, naïve applications of the compressed sensing approach cannot provide success certificates for the decoding results; traditional compressed sensing can provide a sparse solution but cannot exclude the possibility that there exists another solution (possibly denser) fitting the testing results. In addition, different Ct values (which are in nature the logarithm of the viral load) dictate a wide range for viral loads measured in real numbers (for example, from 10^−6^ to 10^6^). Traditional compressed sensing techniques cannot handle such a large range of viral loads accurately and, in basis pursuit decoding results, we cannot distinguish well between negative samples and positive samples with a small but non-negligible viral load [28,29]. This is especially true when we consider a noisy version of this problem, where *f*^−1^(*y*+*Δy*) = *Ax* where *Δy*∈ℝ^*n*^ characterizes the noise occurring in the measurement of Ct values.

Another difference between solving under-determined systems in compressed sensing and those in the virus testing is that the values of *N* and *n* are often small in the later, and large in the former. A large *N* may not be optimal for maintaining high reliability and minimizing the complexity of mixing process in clinical virus testing **(S1 Fig)**. This subtle difference is critical for successful recovery: the commonly used *L*_1_ minimization in compressed sensing may not be able to recover *x* when *N* is small.

### Novel compressed sensing-based virus decoding algorithm

Addressing the challenges mentioned above, we propose a novel algorithm for decoding viral load from pool testing results **(S2 Fig)**. A unique feature of our algorithm is that our algorithm work under both non-adaptive pool testing (using only one PCR run), and adaptive pool testing which allows our algorithm to provide success certificates for decoding results. Our algorithm utilizes quantitative qRT-PCR readings, beyond traditional binary readings, and novel decoding methods to greatly reduce the number of needed tests.

Our testing/decoding protocol is described by Algorithm 1 (main decoding algorithm), which calls Algorithm 2 (determining definitely positive and definitely negative samples) and Algorithm 3 (sparse support set estimation) as subroutines. Algorithm 1 uses Algorithm 2 (determining definitely positive and definitely negative samples) and pool testing results to decode samples into the set of definitely positive samples (*Pos*), the set of definitely negative samples (*Neg*), and the set of samples with undetermined statuses (*U*). Note that our Algorithm 2 provides a success decoding certificate for the samples in *Pos* and *Neg*. Furthermore, Algorithm 1 utilizes compressed sensing and sparsity inspired Algorithm 3 to estimate a sparse support set (namely a small support set) *K* which contain the set of samples which are either definitely positive or highly likely positive. If only non-adaptive testing is allowed, we can simply already declare *K* as the decoded set of positive samples. If adaptive testing is allowed, Algorithm 1 requests new intelligently-designed (designed under the guidance of the decoded sets *Pos*, *Neg*, *U* and *K*) pool testing, and repeat Algorithm 2 of determining definitely positive and definitely negative samples. This testing-decoding process is repeated until all the samples are decoded to be in either the set of positive samples or the set of negative samples, thus providing success decoding certificates for every sample.

Now we discuss how the decoded sets *Pos*, *Neg*, *U* and K can help optimize the design of newly requested pools in adaptive testing and help save the number of tests. The decoded sparse set *K* provided by Algorithm 3 is an “estimate” of the ground-truth set of positive samples, and can be used to greatly reduce the number of needed tests. For example, we can pool the set of samples not belonging to the union of *Pos*, *Neg* and *K* into a single pool for testing: if the testing result is negative (which happens with high probability), that set of samples can all be classified into set *Neg* without needing any further testing for them; if the testing result is positive (which rarely happens), we can test those involved samples individually. For samples in *K* but not in *Pos* after the first round of testing, we perform individual confirmation testing to obtain success certificate for them in the 2^nd^ round of tests in adaptive testing. In fact, if we adopt such testing designs, we can provide success certificates for each sample within at most 3 rounds of testing, while greatly saving the number of needed tests compared with traditional group testing or individual testing.

Algorithm 1 relies on Algorithm 2 to determine definitely positive and negative samples, and to provide decoding success certificates. To do so, in Algorithm 2, we propose to solve a sequence of minimization and maximization pairs for estimating an upper and a lower bound for each element of $x\in R_{+}^{N}$. For *i* = 1,2,⋯,*N*, we solve

$$\min_{x}\;x_{i},$$

$$\mathrm{such}\;\mathrm{that}\;L_{j}\leq {\left(Ax\right)}_{j}\leq U_{j},j\in \mathrm{supp}(p)$$

$$\;\;\;\;{\left(Ax\right)}_{j}=0,j\notin \mathrm{supp}(p)$$

x≥0,

and

$$\max_{x}\;x_{i},$$

$$\mathrm{such}\;\mathrm{that}\;L_{j}\leq {\left(Ax\right)}_{j}\leq U_{j},j\in \mathrm{supp}(p)$$

$$\;\;\;\;{\left(Ax\right)}_{j}=0,j\notin \mathrm{supp}(p)$$

x≥0,

where $L_{j}=f^{-1}(y_{j}^{ub})=f^{-1}(y_{j}+\eta )$ and $U_{j}=f^{-1}(y_{j}^{lb})=f^{-1}(y_{j}-\eta )$ with *η*>0 being a parameter characterizing the noise in Ct value readings and supp(*p*) = {*i*∈{1,2,⋯,*n*}: *p*_*i*_≠0} is the set of indices whose corresponding elements of *p* are nonzero (namely positive pools). After we get the lower bound estimates $x_{lb}^{*}\in R_{+}^{N}$ ($x_{ub}^{*}\in R_{+}^{N}$), we compare each of its elements with an upper bound virus load threshold parameter *ε*_*vlub*_ (*ε*_*vllb*_). If ${\left(x_{lb}^{*}\right)}_{i}>\varepsilon_{vlub}$ (or ${\left(x_{ub}^{*}\right)}_{i}<\varepsilon_{vllb})$, we claim the *i*-th sample of *x* must be positive (or negative). By performing the comparison for each *i*∈[*N*], we can obtain index sets *Pos* and *Neg* which are the index sets of samples which are definitely positive and definitely negative, respectively. Finally, the index set of samples whose status cannot be determined can be obtained as *U*: = [*N*]\(*Pos*∪*Neg*).

Algorithm 3 (sparse support set estimation) is instead used to estimate a “most likely” sparse support set, namely the set of positive samples. Algorithm 3 is inspired by ideas in compressed sensing which takes advantage of the viral load vector often being sparse. However, there is a key difference between traditional compressed sensing and how Algorithm 3 is used in our decoding: traditional compressed sensing cannot guarantee that sparse support set must be the ground-truth set of positive samples; in contrast, Algorithm 1 can utilize Algorithms 2 and 3 to provide eventual success decoding certificates using as few tests as possible, as explained above. To perform “sparse support set estimation”, Algorithm 3 uses previously decoded *Pos*, *Neg*, *U* and solves a weighted least square problem for each possible cardinality *k*∈{1,2,⋯,|*U*∪*Pos*|} and for each possible support set *K*⊆*U*∪*Pos* with cardinality with *k* elements, i.e.,

$$\min_{x}\sum_{j\in \mathrm{supp}(p)}\frac{{\left(f^{-1}(y_{j})-{\left(Ax\right)}_{j}\right)}^{2}}{{\left(f^{-1}(y_{j})\right)}^{2}},$$

$$\mathrm{such}\;\mathrm{that}\;x_{K}\geq 0,x_{(Pos\cup U)\backslash K}=0,x_{Neg}=0.$$

The main idea is to estimate a sparse virus load vector $x\in R_{+}^{N}$ such that the deviation between the estimated pool virus load (*Ax*)_*j*_ and the corresponding interpolated pool virus load *f*^−1^(*y*_*j*_) is minimized. Due to the wide range that the sample virus load can reside, i.e., from 10^−6^ to 10^6^, we normalize the deviation via a scaling factor $\frac{1}{f^{-1}(y_{j})}$. If the solution of the optimization problem above gives a good fit for the pool measurement results, we will update the involved the samples as highly likely positive samples, and include them in the sparse support set.

Usually in practice, the combinatorial characteristics of the exhaustive search can bring high computational complexity and high accuracy. In our virus testing problem, due to the small size of the problem, the exhaustive search can be a good option. Another technique we use to reduce the computational complexity is that we try to find the sparsest solution. This is achieved by finding the solution with the smallest support set such that the misfit between the estimated Ct value and the measured Ct value is smaller than a given tolerance for all the observed positive pools.

Overall, in our testing protocol (described by Algorithm 1), after we find a sparsest solution to the testing result, we continue to provide a success certificate for our decoding results. We pool the individual samples that are in *U* but not in the set *K* of highly likely positive samples together into a pool (namely pool the samples which are highly likely to be negative samples but not yet certified to be negative), and test them collectively. If the testing result for that pool is negative, all the involved samples are certified to negative. For the samples in the sparsest solution but not in the set *Pos*, we also test them individually to certify the correctness of the decoding results. In summary, through an adaptive data requesting component (more abstractly detailed below), whenever our protocol decode a sample to be positive or negative, that decoding result comes with a success certificate.

The adaptive data requesting component is motivated by the fact that even though one round of pooling tests is usually enough to accurately determine the status of all the individuals, extra rounds of pooling can be requested if a single round of pooling test is not enough to provide decoding success certificate. In this scenario, we denote the mixing matrix *A* in the first round by $A^{\left(1\right)}\in R_{+}^{n_{1}\times N}$ where *n*_1_ = *n*, and the mixing matrices in the later rounds by $A^{\left(2\right)}\in R_{+}^{n_{2}\times N}$, $A^{\left(3\right)}\in R_{+}^{n_{3}\times N}$, ⋯. Correspondingly, the pooling results in different rounds are $y^{\left(1\right)}\in R_{+}^{n_{1}}$, $y^{\left(2\right)}\in R_{+}^{n_{2}}$, ⋯ where *y* = *y*^(1)^. Then in the *i*-th round of pooling, we use all the pooling results $y^{\left(1\right)}\in R_{+}^{n_{1}}$, $y^{\left(2\right)}\in R_{+}^{n_{2}}$, ⋯, $y^{\left(i\right)}\in R_{+}^{n_{i}}$ and the mixing matrices $A^{\left(1\right)}\in R_{+}^{n_{1}\times N}$, $A^{\left(2\right)}\in R_{+}^{n_{2}\times N}$, ⋯, $A^{\left(i\right)}\in R_{+}^{n_{i}\times N}$ for estimating all the individual samples. The extra pooled testing results can be obtained for samples whose status and viral load cannot be determined by previous pooled testing results. The mixing matrices for pooling the undetermined samples can be case-specific in practice or follow the pool-highly-likely-negative-sample designs described above in the manuscript. Under the multi-stage pooling framework, a visual illustration of the estimation in the *i*-th stage pooling is presented in **S3 Fig**

### Compressed sensing pooling protocol validation

To demonstrate a proof of concept, we simulated experimental parameters as in Fig 3 where a MATLAB-based script was used to generate pseudorandom experimental parameters based on *N =* 7, 15, and 31 total samples with an overall prevalence rate ranging between 0–10%. The script was ran 25 times for each *N* returning 75 unique testing scenarios. Due to random distribution of positive (ie infected) samples, the individual experiment prevalence rates (defined as the number of positive samples divided by *N*) ranged between 0–14%. We subsequently tested pooling scenarios with the model coronavirus, MHV-1, to generate positive samples in testing scenarios with at least one positive sample per pool [31–36]. Experiments where all samples within a pool were negative were excluded from in lab physical validation testing. Samples were mixed together to form *n* different pools according to the participation matrix in **Fig 2**. Total RNA was extracted from the generated pools utilizing our 1:4 modified pooling technique **(Fig 3A)**. Total RNA isolated from sample pools was then amplified via qRT-PCR to generate a numerical readout of Ct values. To avoid accidental errors, for every group of *N* samples and a given mixing matrix *A*∈ℝ^*n*×*N*^ (where *n is* is *n*_1_ in Algorithm 1) experiments were duplicated. Ct values from the tested pools were delivered to the decoding team blinded to all experimental parameters including the prevalence rate. In all the experiments carried out in this manuscript, we have obtained 100% sensitivity and specificity for our compressed sensing-based approach.

In one of our experiments where *N* = 31, pools 1, 2, 4, and 5 returned Ct values within the bounds to be considered positive **(Table 1)**. With this information alone, Algorithm 2 can decode the samples with ***Neg*** = {**3,6,8,9,12,13,14,15,16,20,21,23,24,25,27,29**} as negative, and the rest of the samples are undetermined. This means ***U*** = {**1,2,4,5,7,10,11,17,18,19,22,26,28,30,31**}, and ***Pos*** = ∅ **(S1 Table)**. These sets are consistent with the ground-truth viral loads in that the samples decoded by Algorithm 2 as negative indeed have almost zero virus load. We remark that, however, our Algorithm 3 of finding a sparse support can significantly reduce the size of the set of samples considered highly likely to be positive. For example, from the decoding results using Algorithm 3, we can see that apart from giving zero estimate for the virus load of samples specified by ***Neg***, Algorithm 3 also estimates all samples from ***U*,** except sample 17, to have zero virus load. This result can be validated with request for one extra pooling test involving all the samples in ***U*** except 17.

**Table 1:** *N* = 31 MHV-1 pooled testing qRT-PCR results.

Pool #	Status	Ct Duplicate 1	Ct Duplicate 2
Pool 1	Pos	35.068	34.234
Pool 2	Pos	35.107	34.526
Pool 3	Neg	NA	NA
Pool 4	Pos	35.021	34.697
Pool 5	Pos	34.031	34.123

**Table 2:** Compressed sensing decoded pooled testing significantly decreases the number of tests required to identify infected samples.

				# of Patient Infection Status Identified				
Number of Patients per Pool	Total Simulated Samples Tested	Total Samples Experimentally Pooled	Experimental Prevalence Rate	Round 1	Round 2	Round 3	Tests Required	% Tests Saved Compared to Individual Testing
7	175	7	0–14.3%	7 (100%)	NA	NA	3	57.10%
15	375	75	0–6.7%	65 (86.7%)	75 (100%)	NA	23	69.30%
31	775	217	0–6.45%	193 (88.9%)	203 (93.5%)	217 (100%)	54	81.40%
Totals:	1325	299	0–14.3%	265 (88.6%)	285 (95.3%)	299 (100%)	80	69.26%

After initial pooling and decoding, further pooling for confirmation testing may be required. We will refer to the matrix in **Fig 2C** as ***P***^(**1**)^. From our decoding result, we request an additional pooling test (***P***^(**2**)^) since not all sample infection statuses can be determined with 100% certainty. Thus, we designed the mixing matrix which pools all the samples that are highly negative **(S4 Fig)**. If the result of that pool comes back negative, we have confirmed that the involved samples are indeed negative; if that pool turns out to be positive (which is highly unlikely to happen), we will request testing the involved samples individually. Viral loads which are very small in magnitude can be due to numerical error, and we can simply treat it as 0.

In total, 80 tests were required to identify all positive samples within the population of 299 total samples. After a single round of testing, the infection status of 88.6% of all samples was established with 100% certainty regardless of prevalence rate or pool size. One subsequent round of verification testing identified the infection status of 95.3% samples and 14 remaining samples which required further testing to determine infection status with full certainty. This resulted in a 69.26% reduction in the total number of tests required as compared to individualized testing. These experiments were repeated with similar parameters and results bringing the total number of computer simulated samples tested to 2650 and experimentally tested samples to 598 **(Table 2)**.

To further validate our pooling and detection system, we obtained human patient RNA samples from the University of Iowa diagnostic testing laboratory. Samples were provided as extracted RNA; thus, our modified RNA extraction protocol was not utilized **(Fig 3A).** An optimized participation matrix was generated to reflect the expected dilution effect **(Fig 2D)**. Experimental parameters were pseudo-randomly generated as previously described with a total *N* of 40 patients and a set prevalence rate of 10%. To compare to currently accepted pooling guidelines (traditional pooling), we used the same experimental parameters to define positive patients within pools containing 5 patients which has been previously determined as optimal for high prevalence rates at or >10% [23]. Pools that were positive were subsequently tested individually, per CDC guidelines, to confirm positivity. The pooling results for one of two independent experiments is presented in Table 3. For both of the two runs, we requested extra pooling results for decoding, and thus required the generation of an additional mixing matrix **(S5 Fig)**. Additional pooling results and individual patient viral loads is shown in **S1 and S2 Tables**.

**Table 3:** Human COVID-19 sample pooled testing qRT-PCR results.

Pool #	Status	Ct Duplicate 1	Ct Duplicate 2
Pool 1	Pos	33.92	32.961
Pool 2	Pos	20.68	20.909
Pool 3	Pos	34.065	36.231
Pool 4	Pos	26.562	27.051
Pool 5	Neg	NA	NA
Pool 6	Pos	26.719	26.864
Pool 7	Pos	19.977	20.386
Pool 8	Pos	27.063	27.756
Pool 9	Neg	NA	NA
Pool 10	Pos	20.636	20.945
Pool 11	Pos	27.574	27.266
Pool 12	Pos	20.196	20.925
Pool 13	Neg	NA	NA
Pool 14	Pos	32.336	32.185
Pool 15	Neg	NA	NA
Pool 16	Pos	33.133	32.97

After one round of testing and compressed sensing decoding, 2 patients were identified and confirmed as positive and 72 were confirmed as negative leaving 6 patients as likely positive **(Table 3)**. Two subsequent pools and four individual confirmation tests provided adequate data points to determine the infection status of all patients with 100% certainty **(S2 Table)**. 32 tests were required to determine the infection status of 92.5% of all patients. Additional confirmatory testing brought the total tests performed to screen 80 patients to 38 **(S3 Table)**. This is a 52.5% reduction in the number of tests needed as compared to current individual testing. Additionally, traditional pooling methods required 51 total tests to identify the status of all 80 patients which is 34.2% more than what was required using our compressed sensing method **(Table 4)**.

**Table 4:** Compressed sensing decoded pooled testing accurately identifies positive human COVID-19 samples at real world prevalence rates.

				# of Patient Infection Status Identified					
	Number of Patients per Pool	Total Patient Samples Pooled	Experimental Prevalence Rate	Round 1	Round 2	Round 3	Tests Required	Tests Required for Traditional Pooling	% Tests Saved Compared to Individual Testing
**Human COVID-19 Swabs**	40	80	10%	74 (92.5%)	80 (100%)	NA	38	51	52.50%

## Discussion

Together, our experimental data provides a proof of concept and validates our compressed sensing pooling technique as an efficient, effective, and reproducible method to greatly increase COVID-19 testing capacity. Using our novel testing approach, we were able to identify positive samples with extreme accuracy at prevalence rates at ≥10% in both an MHV-1 coronavirus model system and with human COVID-19 patient samples. This required approximately one third as many tests as would be needed with current individual testing procedures **(Tables 2 and 4)**.

Pooled testing is an effective approach to increase testing capacity and allow widespread screening to occur and has been implemented with limited success during the COVID-19 pandemic [9–14,40,46–51]. However, current pooled testing efforts lose efficacy and precision as prevalence rates increase and ultimately require substantial additional confirmation testing. Advances in pooled testing have arisen in response to the COVID-19 pandemic where accurate testing of pool sizes of up to 16 individuals has been reported [22]. However, Eberhardt et al. showed the improvement factor over individual testing was dramatically reduced as pool size and prevalence increased requiring multi-stage testing with advantages only at relatively low prevalence rates of 1–5%, well below current COVID-19 rates [22]. In 2020 for the first time in the field, we proposed to use compressed sensing techniques for quantitative virus testing with high prevalence, and computational experiments validated the effectiveness of our method [49]. Others such as Ghosh et al. and Shental et al., showed the superiority of compressed sensing virus testing technology using a non-adaptive approach though their method could only succeed at low prevalence rates <10% [50,51]. In contrast, our current work uses an adaptive approach and can succeed at prevalence rates >10% and utilizes a success certificate to ensure results are accurate.

Current CDC and FDA regulatory guidelines allow for pooled SARS-CoV-2 sample testing, though the number of patients per pool is recommended to stay under 5 and must include viral copy number quantification via a standard curve. However, the CDC states this method provides a testing benefit at prevalence rates <5% and when one individual per pool is positive [23,40,52]. Additionally, strict validation showing the ground truth Ct value of an individual does not increase more than 1.7 cycles after pooling to reduce to risk of sample dilution and false negatives [52]. Abdalhamid et al. reported a pooling method that returned accurate positivity results though they observed that the Ct values increased up to 5.03 cycles due to sample dilution. Due to this dilution effect on the Ct value, they recommended an optimal pool size of 5 individuals [23]. To minimize the pooling dilution effect, we utilized a modified RNA extraction protocol which differs from current clinical diagnostic lab procedures by simply concentrating the RNA to a set volume regardless of the number of input samples **(Fig 3A)**. This standardizes the dilution to an expected and reproducible ΔCt from the ground truth value that does not change if the number of samples within a pool increases **(Fig 3B)**. This protocol alone removes the risk of samples with low levels of virus being diluted in a pool and being read as a false negative. The modified RNA extraction protocol was not a requirement as we were able to accurately and reproducibly identify infected samples in primary human SARS-CoV-2 pooling experiments without utilizing the modified protocol. However, inclusion of this modification to our compressed sensing pooling method increased the ease of identifying infected samples within a pool. This demonstrates the adaptability and flexibility of our compressed sensing pooling method **(Table 2)**.

Our approach demonstrates an effective process to combat testing bottlenecks for future pandemics. Many clinical testing labs currently utilize automated 96-well plate RNA extraction systems in which parameters can be changed to fit our new protocols. We have generated optimized pool mixing matrices at *N* = 7, 15, 31, and 40 demonstrating the adaptability of our approach which can be further adjusted to fit clinical testing needs in multiples of 8 and 12. Additionally, we have created a decoding software in which qRT-PCR data can be entered and the program will decode the data, identify positive samples, and generate additional pools for further testing. Most importantly, the application of our testing method is broad and can be applied to many testing applications within medicine and beyond such as serum antibody testing, drug screening, avian influenza surveillance, water contamination testing, etc. Our compressed sensing testing method is perfectly positioned for testing applications such as these as they are sparse by nature and require accurate results from many data points.

The emergence of new pathogens and variants is ongoing and will continue to be a significant threat to public health and humanity as a whole [4–8]. Implementing a highly accurate pooled testing procedure is absolutely critical to mitigating the spread of viral pandemics such as COVID-19, thus saving lives and decreasing the economic impact from high mortality rates and widespread quarantines. Our use of compressed sensing in pooled testing demonstrated high sensitivity in experimental infection models with the model coronavirus MHV-1, as well as with primary human SARS-CoV-2 samples. The utilization of compressed sensing theory in signal analysis is well established, but its use in the testing of physical specimens has the potential to revolutionize how we provide accurate results when testing large numbers of samples. This will position healthcare professionals to rapidly respond to future pandemics by identifying infected individuals early, minimize spread, and save lives.

## Materials and methods

### Ethics statement

The University of Iowa determined that this project did not meet the regulatory definition of human subjects research and therefore IRB approval was not required.

### Generation of experimental parameters and positive MHV-1 samples

A MATLAB-based computer script was used to generate pseudorandom viral loads for each of *N* individual samples based on an average prevalence rate of 5%, and positive sample Ct values in the range 12–34. The MHV-1 standard curve was used to plot the generated sample Ct value (X) and interpolate the dilution of MHV-1 virus stock (Y) required. According to these estimates *Y*, MHV-1 was diluted in viral transport media as in the CDC-approved nasopharyngeal swab collection protocol [42,44].

### MHV-1 sample pooling

5–20 μL of generated MHV-1 samples were pooled together in equal volumes on ice as designated by the appropriate mixing matrix. Negative samples were added as sterile viral transport media.

### Human patient sample pooling

Human samples that were to be discarded were supplied as extracted RNA in 96-well plates from the University of Iowa Diagnostic Testing Lab. Patients were identified as positive or negative with no information on Ct number, viral load, or any patient identifiable information. 5 μL of patient samples were pooled together in equal volumes on ice as designated by the appropriate mixing matrix.

### Isolation of viral RNA

Viral RNA was extracted via a modified TRIzol phenol/chloroform extraction protocol and can be scaled as needed **(Fig 2)**. A patient pool of 20 μL total volume was mixed with 200 μL TRIzol. The sample was vortexed for 10 sec and incubated for 5 min at room temperature (RT). 40 μL of chloroform was added, vortexed for 10 sec, and incubated for 5 min at RT. The mixture was centrifuged at 12,000 x g for 10 min at 4°C. 100 μL of the upper aqueous layer was transferred to a sterile 1.5 mL tube. 100 μL of isopropanol supplemented with 2 μg glycogen was added, vortexed for 10 sec, and incubated for 5 min at RT. The pellet was mixed with 180 μL of 75% ethanol and resuspended by gentle inversion and centrifuged at 14,000 x g for 10 min at RT. The supernatant was aspirated and the pellet was air dried for 10 min in a sterile laminar flow hood. The RNA pellet was resuspended in 20 μL of RNAse-free diethyl pyrocarbonate-treated H_2_O and incubated at 55°C for 5 min.

### qRT-PCR

5μL of patient pools and samples were mixed with the GoTaq qRT-PCR master mix (Promega) and ran in duplicate on a QuanStudio 3 thermocycler via the FAST qRT-PCR protocol as recommended by the CDC [44]. An MHV-1 virus stock or SARS-Cov-2 S protein containing plasmid of known concentrations were used to generate a standard curve consisting of seven to ten 10-fold serial dilutions. The resulting amplification curves were analyzed with AppliedBiosystems Design and Analysis 2.4.

### Compressed sensing decoding

An optimization algorithm leveraging the non-negativity of viral loads was used to give an upper and lower bound on the viral load for each sample. If the lower bound for a sample’s viral load is not zero, we are sure that that sample is positive; if the upper bound for a sample’s viral load is equal to 0, we are sure that that sample is negative. This identifies samples which are either definitely positive or definitely negative. For the samples with ambiguous infection statuses, we perform exhaustive search for the smallest set of positive samples (namely sparsest solution, having the smallest number of positive samples) fitting the observed viral loads of these pools. The remaining samples were mixed together into a pooled sample to confirm that they are indeed negative: if this pooled sample comes back positive, further testing will be necessary, but this is statistically unlikely. All data, code, and materials are available at: https://github.com/WXU2022/00_CS_Virus_Testing.

## Supporting information

S1 Fig Compressed sensing accuracy increases with *N*. Random test simulation to assess the performance of compressed sensing at low and high *N*. A Bernoulli random matrix *A*∈{0,1}^*n*×*N*^ with *Pr*(*A*_*ij*_ = 0) = *Pr*(*A*_*ij*_ = 1) = 0.5 is used for both cases. We take *n* = round(0.3*N), and the *x* is generated uniformly from [0,100]^*N*^ with sparsity round(0.05*N). The horizontal axis is the index element of x. The vertical axis is the value of the element. **(A)** *N* = 10. **(B)** *N* = 100.
(TIFF)

S2 Fig Compressed sensing decoding algorithms. **(A)** Algorithm 1 virus decoding. **(B)** Algorithm 2 determining positive and definitely negative samples. **(C)** Algorithm 3 exhaustive search for sparse support set.
(TIFF)

S3 Fig Estimation of the *i*-th stage under the framework of multi-stage pooling. (TIFF)

S4 Fig Adaptive request pooling matrix. Pooling matrix designed for additional testing requests. 1 indicates sample is included in the pool. 0 indicates the sample is not included in the pool.
(TIFF)

S5 Fig Human COVID-19 additional testing pooling matrix. Pooling matrix designed for additional testing requests in human COVID-19 samples. *N =* 40 (3x40). 1 indicates patient is included in the pool. 0 indicates the patient is not included in the pool.
(TIFF)

S1 Table MHV-1 individual sample infection status after one round of testing. (DOCX)

S2 Table Human COVID-19 sample second round qRT-PCR results. (DOCX)

S3 Table Human COVID-19 individual patient infection status results. (DOCX)
