# Supplementary figures and images for: Use of compressed sensing to expedite high-throughput diagnostic testing for COVID-19 and beyond
Source: PLoS Comput Biol. 2022 Oct 24;18(10):e1010629. doi: 10.1371/journal.pcbi.1010629 (PMC9632879; doi:10.1371/journal.pcbi.1010629)

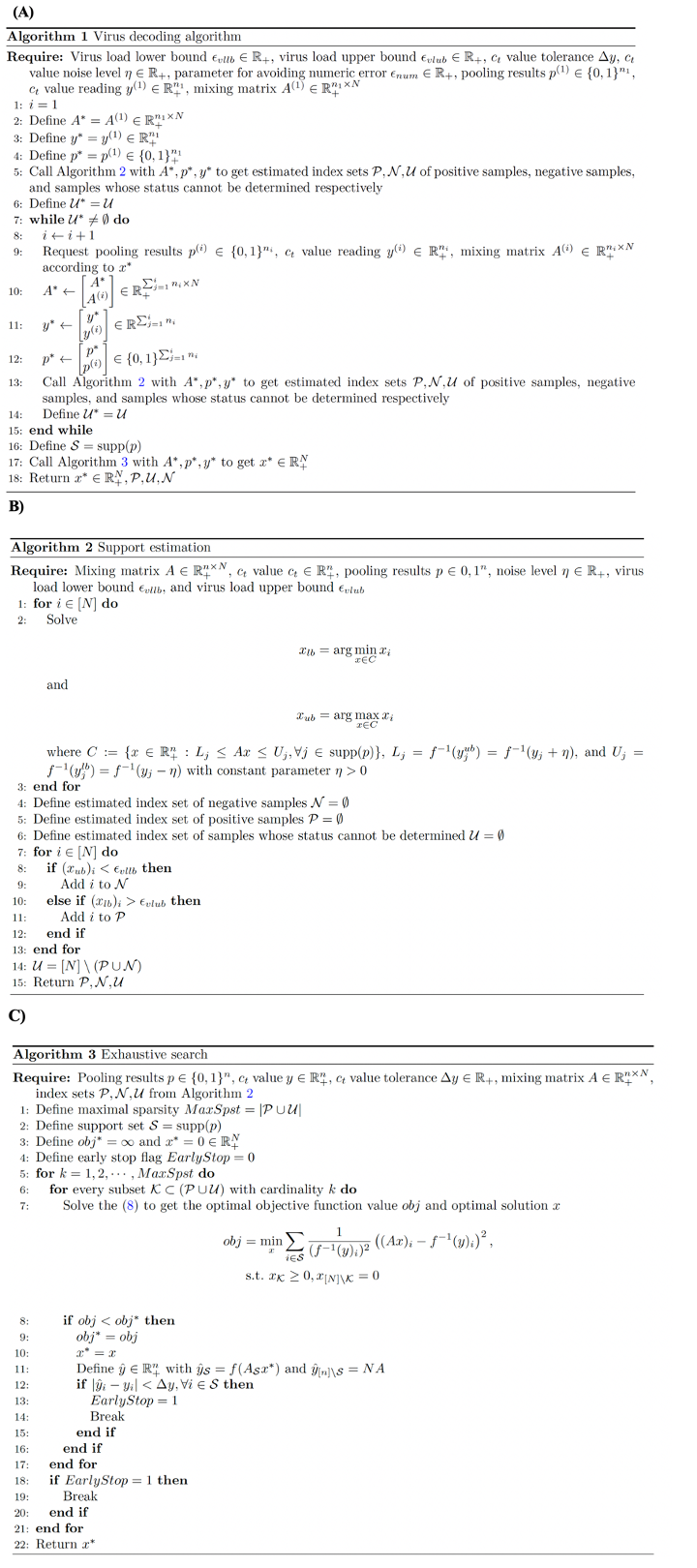

Supplement: S2 Fig — (A) Algorithm 1 virus decoding. (B) Algorithm 2 determining positive and definitely negative samples. (C) Algorithm 3 exhaustive search for sparse support set. (TIFF) [file pcbi.1010629.s002.tiff]

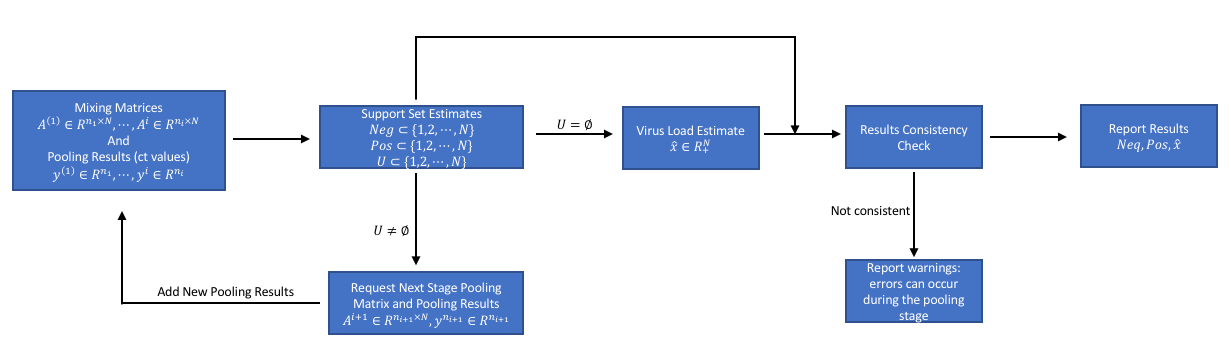

Supplement: S3 Fig — (TIFF) [file pcbi.1010629.s003.tiff]

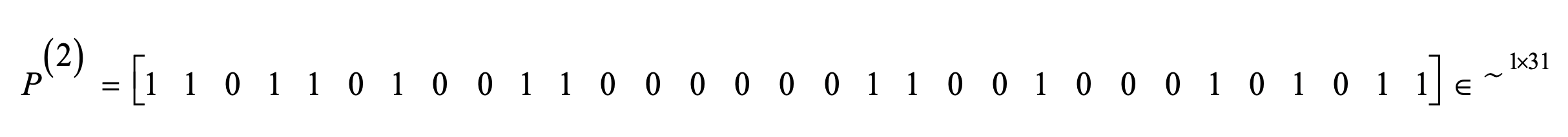

Supplement: S4 Fig — Pooling matrix designed for additional testing requests. 1 indicates sample is included in the pool. 0 indicates the sample is not included in the pool. (TIFF) [file pcbi.1010629.s004.tiff]

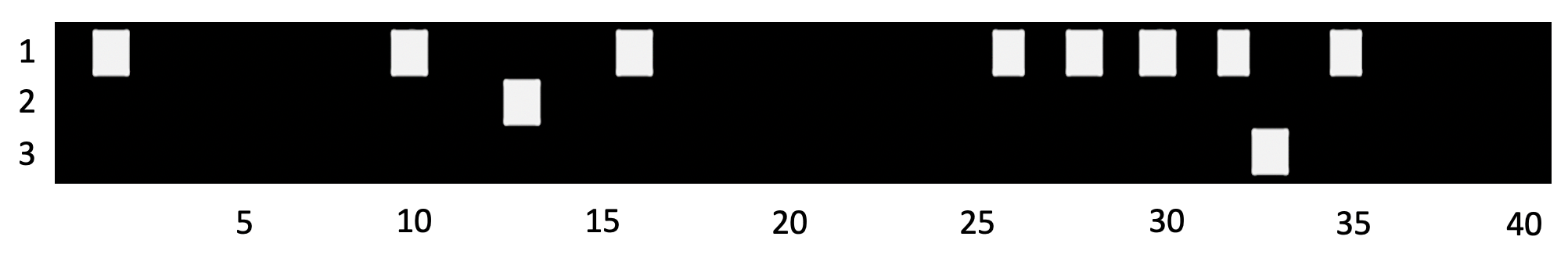

Supplement: S5 Fig — Pooling matrix designed for additional testing requests in human COVID-19 samples. N = 40 (3x40). 1 indicates patient is included in the pool. 0 indicates the patient is not included in the pool. (TIFF) [file pcbi.1010629.s005.tiff]
