# Supplementary material for: Use of compressed sensing to expedite high-throughput diagnostic testing for COVID-19 and beyond
Source: PLoS Comput Biol. 2022 Oct 24;18(10):e1010629. doi: 10.1371/journal.pcbi.1010629 (PMC9632879; doi:10.1371/journal.pcbi.1010629)
Supplement: S1 Table — (DOCX) [file pcbi.1010629.s006.docx]

| **Supplementary Table 1. MHV-1 individual sample infection status** | | | | |
| --- | --- | --- | --- | --- |
| **after one round of testing** | | | | |
|  |  | **Sample Viral Load (ng/mL)** | | |
| **Sample** | **Status** | **Lower Bound** | **Upper Bound** | **Estimated Viral Load** |
| 1 | Undetermined | 2.46E-11 | 3.47E-03 | 0.00E+00 |
| 2 | Undetermined | 2.91E-11 | 3.38E-03 | 0.00E+00 |
| 3 | Negative | 2.50E-11 | 1.15E-12 | 0.00E+00 |
| 4 | Undetermined | 2.61E-11 | 3.57E-03 | 0.00E+00 |
| 5 | Undetermined | 3.31E-10 | 6.77E-03 | 0.00E+00 |
| 6 | Negative | 5.42E-13 | 5.90E-13 | 0.00E+00 |
| 7 | Undetermined | 9.26E-11 | 3.38E-03 | 0.00E+00 |
| 8 | Negative | 5.34E-13 | 5.47E-13 | 0.00E+00 |
| 9 | Negative | 5.32E-13 | 5.63E-13 | 0.00E+00 |
| 10 | Undetermined | 7.74E-11 | 3.38E-03 | 0.00E+00 |
| 11 | Undetermined | 6.24E-11 | 3.47E-03 | 0.00E+00 |
| 12 | Negative | 5.34E-13 | 5.64E-13 | 0.00E+00 |
| 13 | Negative | 5.32E-13 | 5.65E-13 | 0.00E+00 |
| 14 | Negative | 4.97E-13 | 5.78E-13 | 0.00E+00 |
| 15 | Negative | 5.27E-13 | 5.84E-13 | 0.00E+00 |
| 16 | Negative | 5.62E-13 | 6.01E-13 | 0.00E+00 |
| 17 | Undetermined | 7.78E-11 | 3.38E-03 | 1.18E-03 |
| 18 | Undetermined | 7.98E-11 | 3.37E-03 | 0.00E+00 |
| 19 | Undetermined | 9.59E-11 | 3.38E-03 | 0.00E+00 |
| 20 | Negative | 5.42E-13 | 5.93E-13 | 0.00E+00 |
| 21 | Negative | 5.37E-13 | 5.92E-13 | 0.00E+00 |
| 22 | Undetermined | 5.38E-11 | 3.57E-03 | 0.00E+00 |
| 23 | Negative | 5.10E-13 | 5.66E-13 | 0.00E+00 |
| 24 | Negative | 5.43E-13 | 6.06E-13 | 0.00E+00 |
| 25 | Negative | 5.28E-13 | 5.85E-13 | 0.00E+00 |
| 26 | Undetermined | 7.45E-11 | 3.46E-03 | 0.00E+00 |
| 27 | Negative | 5.27E-13 | 5.88E-13 | 0.00E+00 |
| 28 | Undetermined | 7.40E-11 | 3.38E-03 | 0.00E+00 |
| 29 | Negative | 5.11E-13 | 5.56E-13 | 0.00E+00 |
| 30 | Undetermined | 8.88E-11 | 3.47E-03 | 0.00E+00 |
| 31 | Undetermined | 7.05E-11 | 3.38E-03 | 0.00E+00 |
