# Supplementary material for: Use of compressed sensing to expedite high-throughput diagnostic testing for COVID-19 and beyond
Source: PLoS Comput Biol. 2022 Oct 24;18(10):e1010629. doi: 10.1371/journal.pcbi.1010629 (PMC9632879; doi:10.1371/journal.pcbi.1010629)
Supplement: S2 Table — (DOCX) [file pcbi.1010629.s007.docx]

| **Supplementary Table 2. Human COVID-19 sample second round pooling qRT-PCR results** | | | |
| --- | --- | --- | --- |
|  | | | |
| **Pool #** | **Status** | **Ct Duplicate 1** | **Ct Duplicate 2** |
| Pool 1 | Neg | NA | NA |
| Pool 2 | Pos | 29.592 | 30.125 |
| Pool 3 | Neg | NA | NA |
