# Supplementary material for: Use of compressed sensing to expedite high-throughput diagnostic testing for COVID-19 and beyond
Source: PLoS Comput Biol. 2022 Oct 24;18(10):e1010629. doi: 10.1371/journal.pcbi.1010629 (PMC9632879; doi:10.1371/journal.pcbi.1010629)
Supplement: S3 Table — (DOCX) [file pcbi.1010629.s008.docx]

| **Supplementary Table 3. Human COVID-19 individual patient infection** | | | | | |
| --- | --- | --- | --- | --- | --- |
| **status results** | |  |  |  |  |
|  |  | **Sample Viral Load (ng/mL)** | | | |
| **Patient** | **Status** | **Lower Bound** | **Upper Bound** | **Successive Estimated Viral Load** | **Exhaustive Estimated Viral Load** |
| 1 | Negative | 1.52E-13 | 2.45E-14 | 0.00E+00 | 0.00E+00 |
| 2 | Negative | 7.56E-15 | 1.26E-13 | 0.00E+00 | 0.00E+00 |
| 3 | Negative | 3.07E-14 | 8.63E-13 | 0.00E+00 | 0.00E+00 |
| 4 | Negative | 1.60E-13 | 2.30E-14 | 0.00E+00 | 0.00E+00 |
| 5 | Negative | 2.05E-13 | 1.63E-14 | 0.00E+00 | 0.00E+00 |
| 6 | Negative | 9.21E-15 | 1.13E-14 | 0.00E+00 | 0.00E+00 |
| 7 | Negative | 9.30E-14 | 8.24E-15 | 0.00E+00 | 0.00E+00 |
| 8 | Negative | 1.94E-14 | 2.53E-14 | 0.00E+00 | 0.00E+00 |
| 9 | Negative | 1.67E-13 | 1.36E-14 | 0.00E+00 | 0.00E+00 |
| 10 | Negative | 5.68E-14 | 9.01E-15 | 0.00E+00 | 0.00E+00 |
| 11 | Positive | 1.19E+02 | 2.02E+03 | 3.62E+05 | 3.62E+05 |
| 12 | Negative | 2.61E-13 | 1.54E-14 | 0.00E+00 | 0.00E+00 |
| 13 | Positive | 2.57E+02 | 3.21E+03 | 5.63E-02 | 5.63E-02 |
| 14 | Negative | 7.48E-15 | 1.27E-13 | 0.00E+00 | 0.00E+00 |
| 15 | Positive | 2.03E+06 | 2.29E+07 | 6.80E+06 | 6.80E+06 |
| 16 | Negative | 1.36E-13 | 7.69E-15 | 0.00E+00 | 0.00E+00 |
| 17 | Negative | 9.02E-14 | 2.17E-15 | 0.00E+00 | 0.00E+00 |
| 18 | Negative | 2.65E-14 | 7.18E-15 | 0.00E+00 | 0.00E+00 |
| 19 | Negative | 2.94E-14 | 2.43E-14 | 0.00E+00 | 0.00E+00 |
| 20 | Negative | 8.30E-15 | 1.08E-14 | 0.00E+00 | 0.00E+00 |
| 21 | Negative | 1.46E-14 | 2.71E-14 | 0.00E+00 | 0.00E+00 |
| 22 | Negative | 1.56E-13 | 9.77E-15 | 0.00E+00 | 0.00E+00 |
| 23 | Negative | 7.91E-15 | 1.29E-13 | 0.00E+00 | 0.00E+00 |
| 24 | Negative | 7.62E-14 | 7.45E-15 | 0.00E+00 | 0.00E+00 |
| 25 | Negative | 1.28E-14 | 8.53E-15 | 0.00E+00 | 0.00E+00 |
| 26 | Negative | 9.19E-15 | 1.61E-14 | 0.00E+00 | 0.00E+00 |
| 27 | Negative | 2.67E-14 | 1.06E-14 | 0.00E+00 | 0.00E+00 |
| 28 | Negative | 8.88E-15 | 1.21E-13 | 0.00E+00 | 0.00E+00 |
| 29 | Negative | 1.86E-13 | 1.98E-14 | 0.00E+00 | 0.00E+00 |
| 30 | Negative | 1.40E-13 | 8.99E-15 | 0.00E+00 | 0.00E+00 |
| 31 | Negative | 3.46E-15 | 1.86E-15 | 0.00E+00 | 0.00E+00 |
| 32 | Negative | 1.31E-13 | 1.40E-13 | 0.00E+00 | 0.00E+00 |
| 33 | Negative | 5.38E-15 | 7.96E-14 | 0.00E+00 | 0.00E+00 |
| 34 | Negative | 1.32E-14 | 5.13E-15 | 0.00E+00 | 0.00E+00 |
| 35 | Negative | 2.09E-13 | 1.55E-14 | 0.00E+00 | 0.00E+00 |
| 36 | Negative | 7.76E-13 | 1.59E-14 | 0.00E+00 | 0.00E+00 |
| 37 | Negative | 8.14E-15 | 1.03E-13 | 0.00E+00 | 0.00E+00 |
| 38 | Negative | 8.13E-14 | 7.60E-15 | 0.00E+00 | 0.00E+00 |
| 39 | Negative | 1.64E-13 | 2.95E-14 | 0.00E+00 | 0.00E+00 |
| 40 | Positive | 2.43E+04 | 2.21E+05 | 7.52E+04 | 7.52E+04 |
